# The affinity purification and characterization of ATP synthase complexes from mitochondria

**DOI:** 10.1098/rsob.120160

**Published:** 2013-02

**Authors:** Michael J. Runswick, John V. Bason, Martin G. Montgomery, Graham C. Robinson, Ian M. Fearnley, John E. Walker

**Affiliations:** The Medical Research Council Mitochondrial Biology Unit, Hills Road, Cambridge CB2 0XY, UK

**Keywords:** mitochondria, ATP synthase, inhibitor protein, purification, coupling

## Abstract

The mitochondrial F_1_-ATPase inhibitor protein, IF_1_, inhibits the hydrolytic, but not the synthetic activity of the F-ATP synthase, and requires the hydrolysis of ATP to form the inhibited complex. In this complex, the α-helical inhibitory region of the bound IF_1_ occupies a deep cleft in one of the three catalytic interfaces of the enzyme. Its N-terminal region penetrates into the central aqueous cavity of the enzyme and interacts with the γ-subunit in the enzyme's rotor. The intricacy of forming this complex and the binding mode of the inhibitor endow IF_1_ with high specificity. This property has been exploited in the development of a highly selective affinity procedure for purifying the intact F-ATP synthase complex from mitochondria in a single chromatographic step by using inhibitor proteins with a C-terminal affinity tag. The inhibited complex was recovered with residues 1–60 of bovine IF_1_ with a C-terminal green fluorescent protein followed by a His-tag, and the active enzyme with the same inhibitor with a C-terminal glutathione-*S*-transferase domain. The wide applicability of the procedure has been demonstrated by purifying the enzyme complex from bovine, ovine, porcine and yeast mitochondria. The subunit compositions of these complexes have been characterized. The catalytic properties of the bovine enzyme have been studied in detail. Its hydrolytic activity is sensitive to inhibition by oligomycin, and the enzyme is capable of synthesizing ATP in vesicles in which the proton-motive force is generated from light by bacteriorhodopsin. The coupled enzyme has been compared by limited trypsinolysis with uncoupled enzyme prepared by affinity chromatography. In the uncoupled enzyme, subunits of the enzyme's stator are degraded more rapidly than in the coupled enzyme, indicating that uncoupling involves significant structural changes in the stator region.

## 2. Introduction

The F-ATP synthase, or F_1_F_o_-ATPase, from mitochondria is an abundant multi-subunit assembly associated with the inner membranes of the organelle [1,2]. The high-resolution structural analysis of the F-ATP synthase has been conducted by the detailed analysis of constituent domains, largely by X-ray crystallography [3–10]. These substructures have been assembled into an overall mosaic structure within the constraints of a 32 Å resolution overall structure determined by cryo-electron microscopy of single particle images of the intact enzyme complex [10,11]. A series of structures of the F_1_ catalytic domain from both the bovine [3–7,12] and yeast [13,14] enzymes describes the structural changes that occur in the α_3_β_3_ subdomain in response to the anticlockwise rotation of the central stalk of the enzyme (as viewed from its membrane domain), and provides a detailed description of the catalytic mechanism of ATP hydrolysis by F_1_-ATPase. The structure of the membrane extrinsic region of the enzyme was completed by the addition of the structure of the peripheral stalk, which demonstrated its mode of attachment to the F_1_ domain via an interaction between the oligomycin sensitivity conferral protein (OSCP) with the N-terminal region of one of the three α-subunits [8,9,15]. The structural analysis of the membrane domain of the enzyme is less advanced, but structures have been determined for the F_1_–c ring complexes from bovine [10] and yeast mitochondria [16], which contain the complete rotors. However, as yet, there is no high-resolution structural information describing the rest of the membrane domain of the enzyme, and therefore a molecular description is lacking of how the transmembrane proton-motive force is coupled to ATP synthesis, and of how rotation is generated. One possible approach to filling this lacuna would be to crystallize the intact F_1_F_o_-ATPase complex, and to determine its high-resolution structure by X-ray crystallography.

One severe practical problem especially has impeded this approach. As described in this study, the interface between the c-ring and subunit a is unstable, and the removal of phospholipids and their replacement by detergents often uncouples the proton-motive force from ATP synthesis by disrupting the interactions between subunit a and the c-ring. Thus, any structural information about how they interact is lost. To overcome this problem, as described here, we have developed a simple, rapid and mild purification of the F_1_F_o_-ATPase complex from mitochondria that takes advantage of the exquisitely specific inhibition of the mitochondrial F_1_F_o_-ATPase by its natural inhibitor protein, IF_1_ [17]. The binding of the inhibitor to the enzyme requires the hydrolysis of ATP, and only the ATP hydrolytic (and not its synthetic activity) is inhibited. It has been suggested that IF_1_ may also inhibit ATP synthesis [18], but this effect has not been demonstrated directly on the isolated enzyme. Bovine IF_1_ is a predominantly α-helical protein [19] that, in the inhibited complex, is deeply buried in a channel in one of three catalytic interfaces of the F_1_ catalytic domain of the enzyme [20,21]. In the inhibited complex, the C-terminal part of the inhibitor protein is exposed, and extends from the surface of the F_1_ domain. Therefore, affinity tags have been attached to the C-terminus of appropriately engineered inhibitor proteins to facilitate the purification of the inhibited complex. After the inhibition of the F_1_F_o_-ATPase, the detergent-solubilized inhibited complex has been bound selectively to an appropriate affinity column. Then, in a subsequent step, either the inhibited F_1_F_o_ complex or the active complex has been recovered. These enzyme preparations are free from contaminants, and they are almost entirely fully coupled. The versatility of the procedure is demonstrated here by the purification of the enzyme complex from bovine, ovine, porcine and yeast mitochondria.

## 3. Material and methods

### 3.1. Analytical procedures

Protein concentrations were measured by the bicinchoninic acid method (Pierce Biotechnology). The ATPase activity of samples was determined by coupling it to the oxidation of NADH monitored at 340 nm [22]. In this assay, hydrolysed ATP is regenerated by a transfer of phosphate from phosphoenolpyruvate to ADP catalysed by pyruvate kinase generating pyruvate. Pyruvate is converted to lactate-by-lactate dehydrogenase with concomitant oxidation of NADH to NAD^+^. Thus, the rate of the decrease in absorbance at 340 nm is directly proportional to the rate of ATP hydrolysis. The enzymes and substrates are added in excess to ensure that the rate of NADH oxidation is limited only by the hydrolytic activity of the ATPase. The effect of oligomycin on this activity was determined by addition of the inhibitor (0.1 mg ml^−1^; w/v) in ethanolic solution.

### 3.2. Isolation of mitochondria and mitochondrial membranes

Mitochondria were isolated from bovine, ovine and porcine hearts, as described previously for bovine mitochondria [23], and stored at −20°C. Mitochondria were prepared from 55 l cultures of *Saccharomyces cerevisiae* (W303–1A, Mat *α, ade2-1, trp1-1, leu2-3,112, ura3-1, his3-11,15, ybp1-1* plus a canavanine-resistance marker) grown at 30°C in a medium consisting of peptone (20 g l^−1^), yeast extract (10 g l^−1^), 3 per cent glycerol (v : v), adenine (0.05 g l^−1^) and antifoam 204 (180 μl l^−1^; Sigma-Aldrich) in an Applikon ADI1075 fermentor (Applikon Biotechnology). At the end of logarithmic growth when the OD600 had reached 8–9, the cells were cooled to 20°C, harvested by continuous centrifugation at 18 000*g*, broken by passage through a dyno-mill disruptor (WA Bachofen AG), and centrifuged for 20 min at 4800*g* and then for 10 min at 4200*g*. The mitochondria were obtained from the supernatant by centrifugation (32 000*g*, 50 min). They were washed twice in a buffer containing 100 mM Tris–HCl, pH 7.5, 650 mM sorbitol, 5 mM aminohexanoic acid, 5 mM benzamidine and 0.005 per cent PMSF (w/v), and stored at −20°C, at a protein concentration of 10 mg ml^−1^, in suspension in a buffer consisting of 20 mM Tris–HCl, pH 8.0, containing 10 per cent glycerol (v/v). The yield of mitochondrial protein from a 55 l culture was 6–8 g. Ammonium sulphate (AS) particles were prepared from bovine mitochondria, as described previously [24].

### 3.3. Over-expression and purification of inhibitor proteins

Sequences encoding residues 1–60 of bovine IF_1_ plus C-terminal hexahistidine, and with C-terminal glutathione-*S*-transferase (GST), or green fluorescent protein (GFP), plus hexahistidine, and of residues 14–60 of bovine IF_1_ with C-terminal GST plus hexahistidine, were all cloned individually into the expression plasmid pRun [25]. The proteins were expressed in *Escherichia coli* C41 (DE3), and purified by affinity chromatography on a Hi-Trap nickel sepharose column (5 ml; GE Healthcare), as described previously [26]. Pooled fractions containing inhibitor proteins were dialysed for 4 h against 2 l of buffer consisting of 20 mM Tris–HCl, pH 7.4, and concentrated to 10 mg ml^−1^ with a VivaSpin concentrator (molecular weight cut-off 5 kDa; Sartorius). The yields of inhibitor proteins referred to as I1–60His, I1–60GFPHis and I1–60GSTHis were 10, 100 and 100 mg l^−1^, respectively.

### 3.4. Purification of inhibited F_1_F_o_-ATPase complexes

Bovine heart (and ovine and porcine heart) mitochondrial membranes were suspended in phosphate buffer consisting of 50 mM disodium hydrogen orthophosphate, pH 9.2, 100 mM sucrose and 0.5 mM EDTA, and then centrifuged (13 700*g*, 30 min, 4°C). This procedure, which was repeated twice, removed endogenous IF_1_ bound to the bovine ATPase, but it was not applied to mitochondria from *S. cerevisiae* as they have low amounts of bound endogenous IF_1_. The pellet of phosphate-washed animal mitochondria (or unwashed yeast mitochondria) was re-suspended at a protein concentration of 8.5 or 10 mg ml^−1^, respectively, in a buffer containing 20 mM Tris–HCl, pH 8.0, and 10 per cent glycerol (v/v). To 50 ml portions of this suspension, 5.5 ml of a solution of 10 per cent (w/v) dodecylmaltoside (DDM) was added to a give a final detergent concentration of 1 per cent (w/v). The suspensions were kept at room temperature for 10 min, and then centrifuged (24 000*g*, 10 min). In a typical experiment, the ATPase activity in a DDM extract of bovine mitochondrial membranes (382 mg of protein) was inhibited with I1–60His (1.0 mg), and 750 μl of a solution containing 200 mM ATP, 200 mM MgCl_2_ and 400 mM Trizma base was added. The sample was incubated at 37°C for 15 min, and further portions (750 µl) of the ATP solution were added every 5 min. After centrifugation (10 000*g*, 10 min), sodium chloride (0.3 g) and 5 M neutralized imidazole were added to the supernatant to final concentrations of 0.1 M and 25 mM, respectively. This solution was applied at a flow rate of 1 ml min^−1^ to a nickel Sepharose HisTrap HP column (5 ml; GE Healthcare) equilibrated in buffer B (20 mM Tris–HCl, pH 7.4, 10% (v/v) glycerol, 0.1% (w/v) DDM, 1 mM ATP, 2 mM MgSO_4_, 0.1 M NaCl and 25 mM imidazole). The F_1_F_o_–I1–60His inhibited complex was eluted with a linear imidazole gradient of 25–500 mM over 100 ml. The recovery of the inhibited complex was 25 mg. This procedure was performed at 23°C.

### 3.5. Purification of active F_1_F_o_-ATPase

Active bovine F_1_F_o_-ATPase was purified in a similar way to the inhibited complex, except that the ATPase activity of the DDM extract (50 ml) was inhibited with 2.9 mg of I1–60GSTHis. The F_1_F_o_-I1–60GSTHis complex was applied to two GSTrap HP columns (each 5 ml; GE Healthcare) connected in series, equilibrated in buffer C consisting of 20 mM Tris–HCl, pH 7.3, 0.1 per cent DDM, 10 per cent glycerol, 0.15 M NaCl and 5 mM dithiothreitol. The bound protein was washed with buffer D containing 20 mM Tris–HCl pH 7.3, 0.1 per cent DDM, 10 per cent glycerol and 10 mM EDTA at a flow rate of 1 ml min^−1^. When the conductivity of the eluate had reached a stable baseline, the flow of buffer was stopped for 17 h. Then, the active F_1_F_o_-ATPase complex was recovered at a buffer flow rate of 0.5 ml min^−1^.

The preparation of the active bovine complex was repeated with various phospholipids (final concentration of 0.1 mg ml^−1^) in the chromatography buffers. The phospholipids were asolectin (a mixture of phopholipids from soya beans; Sigma-Aldrich), and the following mixtures (Avanti Polar lipids): bovine cardiolipin (CL) : bovine phosphatidylcholine (PC) : bovine phosphatidylethanolamine (PE) (3 : 1 : 1, by wt); asolectin : bovine CL (1 : 3, by wt); bovine heart polar phospholipid extract (BHPPE); and 1-palmitoyl-2-oleoyl-sn-glycero-3-[phospha-rac-(1-glycerol)] (POPG) : 1-palmitoyl-2-oleoyl-sn-glycero-3-phosphoethanolamine (POPE) : 1-palmitoyl-2-oleoyl-sn­-glycero-3-phosphocholine (POPC) (3 : 1 : 1, by wt).

### 3.6. Partial trypsinolysis of F_1_F_o_-ATPase

Trypsin (3.5 μl; 1 mg ml^−1^ in 1 mM hydrochloric acid) was added to a sample of bovine F_1_F_o_-ATPase (350 μl, 2 mg ml^−1^) in 20 mM Tris–HCl, pH 7.4, containing DDM (0.3%; w/v) to give a trypsin : F_1_F_o_-ATPase ratio of 1 : 200 (w/w). Samples (50 μl) were removed at various intervals up to 360 min, and proteolysis was terminated by addition of a fivefold excess (w/w) of bovine pancreatic trypsin inhibitor (5 mg ml^−1^ in water).

### 3.7. Protein analysis

Samples of mitochondria, mitochondrial membranes, purified F_1_F_o_-ATPases and products of partial trypsinolysis of bovine F_1_F_o_-ATPase were analysed by SDS–PAGE in 10–15 per cent acrylamide gradient gels, and the proteins were detected with Coomassie blue dye. Proteins in stained bands were identified by mass mapping of tryptic peptides [27]. Partial proteolysis products of coupled and uncoupled samples of bovine F_1_F_o_-ATPase were precipitated with ethanol, the subunits of F_1_F_o_-ATPases and the products of partial proteolysis of bovine F_1_F_o_-ATPase were separated by RP-HPLC and their intact protein masses were measured ‘online’ to the column with a Quatro Ultima triple quadrupole mass spectrometer with electrospray ionization (Micromass), as described previously [28].

### 3.8. Preparation of liposomes

Chicken egg phosphatidylcholine and *E. coli* polar lipid extract dissolved in chloroform were mixed in a ratio of 1 : 3 (w : w). The lipid composition was chosen in order to maximize coupling in the liposomes. The particular phospholipid mixture does not compare with the lipids present in the mitochondrial membrane but has been used extensively before in reconstitution studies [29]. The solvent was evaporated in a stream of nitrogen, and the dried phospholipids were re-dissolved in an equivalent volume of water. Unilamellar liposomes of uniform size were prepared by a passage of the solution five times through a polycarbonate filter (0.1 μm pore size; Millipore Corporation). They were stored at 4°C at a phospholipid concentration of 20 mg ml^−1^ in a buffer containing 20 mM MOPS, pH 7.4 and 50 mM KCl.

### 3.9. Reconstitution of F_1_F_o_-ATPase into liposomes

Liposomes (400 μl) were de-stabilized in the presence of samples of purified bovine F_1_F_o_-ATPase (70 μl, 10 mg ml^−1^) by addition of Triton X-100. F_1_F_o_-ATPase was purified in the absence of any phospholipids. The requisite amount of Triton X-100 was calculated from the absorption of the vesicles at 600 nm following addition of 400 μl of phospholipid vesicles to 10 μl quantities up to 50 μl of 10 per cent (w/v) Triton X-100. The volume of the mixture was adjusted to 2.4 ml with 20 mM Tris–HCl, pH 7.4. The detergent was removed by the gradual addition of Biobeads (Biorad Laboratories) up to 10 mg mg^−1^ of detergent and then over 12 h up to a total of 20 mg of Biobeads per mg of detergent. The proteoliposomes were centrifuged (60 000*g*, 45 min) and then re-suspended in buffer (250 μl) containing 20 mM Tris–HCl, pH 7.4.

### 3.10. Proton pumping coupled to ATP hydrolysis in proteoliposomes

A portion of proteoliposomes (10 μl) were added to a solution containing 20 mM MOPS, 20 mM KCl and 0.5 μM valinomycin to give a final volume of 1 ml. 9-amino-6-chloro-2-methoxyacridine (ACMA) was added to a final concentration of 1 μM. The proton-pumping activity of the F_1_F_o_-ATPase was initiated by the addition of ATP to a final concentration of 100 μM, and terminated by the addition of either I1–60GFP or oligomycin, to final concentrations of 0.5 or 0.665 μM, respectively. The change in fluorescence at excitation and emission wavelengths of 430 and 475 nm, respectively, was measured in a Shimaduzu RF-5301 PC dual wavelength spectrophotometer. ACMA binds to membranes in the energized state and becomes quenched when a pH gradient is established.

### 3.11. ATP synthesis in proteoliposomes

Purple membranes were purified from *Halobacterium halobium* as described before [30], and bacteriorhodopsin was solubilized in 2 per cent (v/v) Triton X-100. Proteoliposomes containing both F_1_F_o_-ATPase and bacteriorhodopsin were prepared as described earlier, except that solubilized bacteriorhodpsin (300 μl; 2 mg ml^−1^) was added also to the reconstitution mixture. A portion of the resulting proteoliposomes (10 μl) was suspended in a solution containing 20 mM Tris–HCl, pH 7.4, 200 mM phosphate and 200 mM ADP. The stirred suspension (750 μl) was illuminated with a halogen bulb. Samples (75 μl) were removed at various times and quenched with 75 μl of aqueous trichloroacetic acid (40 g l^−1^). The ATP content was estimated by luciferin–luciferase assay with an ATP Bioluminescence kit (Roche).

## 4. Results

### 4.1. Purification of inhibited and active F_1_F_o_-ATPase

**Figure 1.**
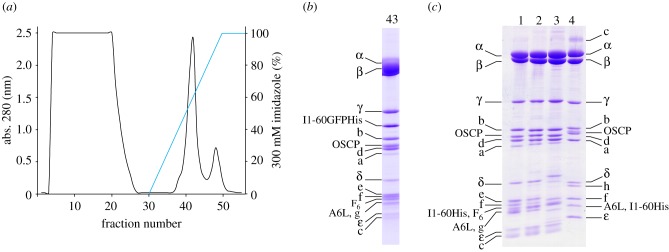
Purification of the F_1_F_o_-ATPase inhibitor complexes from bovine, ovine, porcine and yeast mitochondria by nickel affinity chromatography. The inhibitor was (*a*,*b*) I1–60GFPHis and (*c*) I1–60His. (*a*) Elution profile of the inhibited bovine F_1_F_o_-ATPase-I1–60GFPHis complex monitored at 280 nm. (*b*) SDS–PAGE analysis of fraction 43 (1 ml) from (*a*). The inhibitor protein is found between the γ- and b-subunits (compare with (*c*)). (*c*) Tracks 1–4 are purified inhibited F_1_F_o_-ATPase-I1–60His from cows, sheep, pigs and *S. cerevisiae*, respectively. Subunits were identified by mass spectrometric analysis of peptides derived from stained bands.

The F_1_F_o_-ATPase inhibitor complex was purified from mitochondria from cows, sheep, pigs and *S. cerevisiae* (figure 1). The average yields of the purified complexes from about 380 mg of total protein in phosphate-washed mitochondria were 25, 10 and 16 mg for the bovine, porcine and ovine complexes, respectively. The yield of the inhibited complex from *S. cerevisiae* mitochondria was 15 mg g^−1^ of mitochondrial protein.

**Figure 2.**
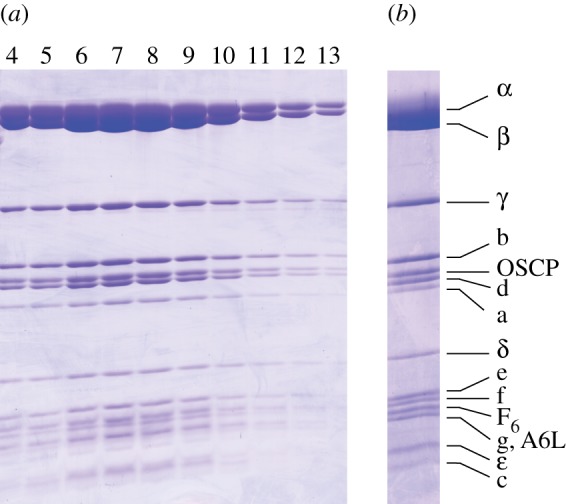
Purification of active bovine F_1_F_o_-ATPase by GST affinity chromatography. (*a*) SDS–PAGE analysis of fractions 4–13 (each 1 ml) recovered from the column after reversal of inhibition with EDTA. (*b*) Analysis of pooled fractions of purified bovine F_1_F_o_-ATPase. Subunits were identified by mass mapping of tryptic peptides derived from stained bands.

The inhibition of mitochondrial F_1_-ATPase by IF_1_ has been reported to be reversed by a range of reagents [31–33]. They include oxyanions such as sulphate, sulphite, bicarbonate, borate, phosphate and pyrophosphate, and the chelating agent EDTA. In an initial survey conducted in solution, each of them was investigated, at various concentrations and under a variety of conditions, as agents for releasing F_1_F_o_-ATPase from the inhibited bovine complex. From these investigations, EDTA was identified as the reagent that released the highest activity of F_1_F_o_-ATPase. It probably acts by removal of magnesium ions associated with nucleotides bound in the catalytic and non-catalytic sites of the enzyme, resulting in the destabilization of the interaction with the inhibitor protein. However, EDTA could not be used for releasing the active enzyme from the inhibited complex bound to a nickel–NTA column via the histidine tag of the inhibitor protein, although borate was used successfully to release the active enzyme (data not shown). Therefore, the inhibited complex was bound to a column of immobilized glutathione via the GST domain of I1–60GSTHis, and then the active enzyme was released with EDTA (figure 2). The recovery of active bovine F_1_F_o_-ATPase from about 380 mg of protein in the DDM extract of mitochondria was 11 mg, with a specific activity of 4.0 µmol min^−1^ mg^−1^.

### 4.2. Subunit compositions of purified F_1_F_o_-ATPase complexes

The subunit compositions of the various purified complexes were analysed by SDS–PAGE, and by mass-mapping the tryptic digests of the bands from the stained gels (figures 1 and 2; electronic supplementary material, tables S1 and S2). These analyses demonstrated that the known complement of subunits of the enzyme was present in the purified bovine-, sheep- and pig-inhibited complexes, and in the active bovine complex, except for the membrane subunits DAPIT and 6.8 kDa proteolipid [34]. It is known that these two subunits are retained in the complex only when the enzymes are purified with phospholipids in the buffers throughout the preparation. The yeast genome does not encode subunits that are equivalent to DAPIT and the 6.8 kDa proteolipid, and the purified yeast enzyme also lacked the known subunits e, g and k, which, although they were retained when a Triton X-100 extract of mitochondria from *S. cerevisiae* was fractionated by anion exchange chromatography [35], are weakly associated with the complex. The separation of the subunits of the enzymes from all four species by liquid chromatography (LC), coupled to measurement of their intact masses by mass spectrometry, confirmed their subunit compositions, and also confirmed the presence of the hydrophobic subunits a and c, which contain few tryptic cleavage sites and so are not readily identifiable by mass mapping of tryptic peptides (see the electronic supplementary material, table S1).

### 4.3. Oligomycin sensitivity of purified F_1_F_o_-ATPase

Oligomycin inhibits ATP hydrolysis and synthesis, and is thought to bind at the interface between the c-ring and subunit a, and prevent rotation [36,37]. The sensitivity of ATP hydrolysis by the enzyme to oligomycin indicates the extent to which ATP hydrolysis is coupled to proton translocation through the membrane domain. When active bovine F_1_F_o_-ATPase was purified in the presence of detergent (DDM) only, the sensitivity of ATP hydrolysis to oligomycin was severely reduced (table 1). This loss of oligomycin sensitivity was decreased to a greater or lesser extent by the incorporation of various phospholipids into the buffers used during affinity purification. The most effective was a mixture of synthetic analogues of PE, PC and PG, with 90 per cent of the hydrolytic activity being sensitive to the inhibitor, and a mixture of CL, PC and PE was almost equally effective (table 1). It should be noted that the inner mitochondrial membrane comprises about 40–50 per cent PC and PE, and approximately 15 per cent cardiolipin [38,39]. However, a greater percentage of cardiolipin was required in order to achieve a high level of oligomycin sensitivity in the purified enzyme (table 1).

**Table 1.** Effect of phospholipids on the activity of bovine F_1_F_o_-ATPase. The phospholipids were added to buffers used in the purification of the active enzyme (see §3).

preparation	additives	specific activity (µmol min^−1^ mg^−1^)	oligomycin sensitivity (% inhibition)
AS particles	none	30.0 ± 3.7^a^	91.0 ± 1.5
DDM extract	none	27.6 ± 4.0^a^	83.8 ± 4.7
F_1_F_o_-ATPase	none	4.4 ± 1.3	8.6 ± 3.1
F_1_F_o_-ATPase	asolectin	50	40
F_1_F_o_-ATPase	CL : PC : PE^b^	22.2 ± 4.8	77.2 ± 3.6
F_1_F_o_-ATPase	asolectin : CL^c^	49	75
F_1_F_o_-ATPase	BHPPE	16	66
F_1_F_o_-ATPase	POPC : POPG : POPE^d^	21.1 ± 3.5	90.0 ± 1.6
F_1_F_o_-ATPase	PL vesicles^e^	24.7	83

^a^10% of the membrane protein was assumed to be F_1_F_o_-ATPase.

^b^3 : 1 : 1, by wt.

^c^1 : 3 w/w.

^d^3 : 1 : 1, by wt.

^e^Phospholipid (PL) vesicles are composed of chicken egg phosphatidylcholine and *E. coli* polar lipid (1 : 3, w/w).

In addition, enzyme prepared in the complete absence of phospholipids has a large increase in oligomycin sensitivity and ATP hydrolysis activity upon reconstitution into phospholipid vesicles (table 1). Therefore, any decrease in coupling and enzyme activity arising from removal of lipid is not completely irreversible.

### 4.4. Comparison of uncoupled and coupled F_1_F_o_-ATPase by limited proteolysis

**Figure 3.**
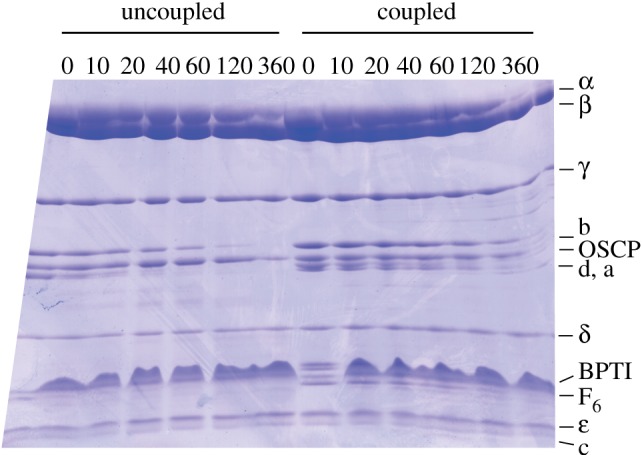
A comparison of the degradation of uncoupled and coupled samples of bovine F_1_F_o_-ATPase by mild proteolysis. The uncoupled and coupled samples correspond to samples of the enzyme purified in the absence of phospholipids and in the presence of synthetic phospholipids, respectively (table 1). Samples were removed after various times (given in minutes across the top of the gel), and the products were analysed by SDS–PAGE. The migration positions of the subunits of the enzyme complex are indicated on the right-hand side of the gel. BPTI is the bovine pancreatic trypisin inhibitor protein used to terminate proteolysis.

**Figure 4.**
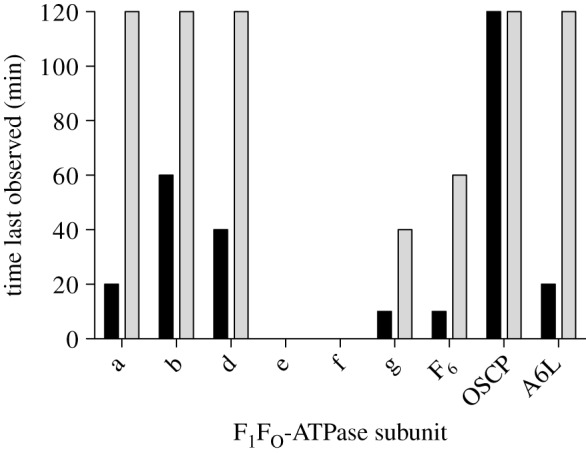
A comparison of the susceptibility to trypsinolysis of the peripheral stalk and F_o_-subunits of bovine F_1_F_o_-ATPase in samples of the uncoupled and coupled enzymes. Samples were analysed by liquid chromatography linked online to mass spectrometry (LC–MS) at various times during a 2 h period of proteolysis. The histograms show the times at which the various subunits, shown on the abscissa, were last observed. Uncoupled and coupled samples of F_1_F_o_-ATPase are shown in black and light grey, respectively.

Structural differences between uncoupled and coupled preparations of active bovine F_1_F_o_-ATPase were probed by limited proteolysis with trypsin. Coupled enzyme (oligomycin sensitivity of 90%) was prepared in the presence of phospholipids (POPC : POPG : POPE, 3 : 1 : 1, by wt), whereas uncoupled enzyme (oligomycin sensitivity of 6%) was prepared in the absence of phospholipids. In these experiments, their patterns of degradation resulting from mild exposure to trypsin over a period of 3 h were compared. Analysis of the products of degradation by SDS–PAGE (figure 3) demonstrated that the peripheral stalk subunits b, d and F_6_ were degraded in the uncoupled enzyme, whereas they were resistant to trypsinolysis in the coupled enzyme. The subunits of the F_1_ domain and the OSCP component of the peripheral stalk, which interacts directly with the F_1_ domain, were resistant to proteolysis in both samples. Subunit c also resisted proteolysis, but this observation is unsurprising as the C-terminal α-helices of the subunit, which form the external exposed surface of the assembled c-ring, contain few trypsin cleavage sites. In both samples, the small membrane subunits, e, f, g and A6L, were obscured in the gel by the bovine pancreatic trypsin inhibitor, and the F_o_ component subunit a, which stains poorly with the Coomassie blue dye, was difficult to discern. Therefore, in order to be able to examine the effect of proteolysis more comprehensively, samples of proteolysis products taken at the various time-points were separated by LC and analysed by mass spectrometry (see the electronic supplementary material, figures S1–S4). In the electronic supplementary material, tables S3 and S4, the results of these analyses are presented as a summary of when the intact subunits in the uncoupled and coupled enzymes were last observed during the 3 h period of proteolysis, when the various specific fragments resulting from proteolysis were first detected and when they were last observed. The data for the first 2 h of degradation of the intact peripheral stalk and F_o_ subunits from uncoupled and coupled enzymes are compared in figure 4. Figure 4 confirms that the peripheral stalk subunits b, d and F_6_ are more susceptible to proteolysis in the uncoupled enzyme than in the coupled enzyme, and a similar trend was found with the F_o_ subunits, a, g and A6L. The F_o_ subunits e and f were degraded rapidly in both samples, and the intact proteins were not detected after 10 min of proteolysis, implying that they are similarly exposed in both the coupled and uncoupled preparations. Figure 4 also confirms that the OSCP subunit is resistant to proteolysis.

### 4.5. Proton-pumping and ATP synthesis activities of bovine F_1_F_o_-ATPase

**Figure 5.**
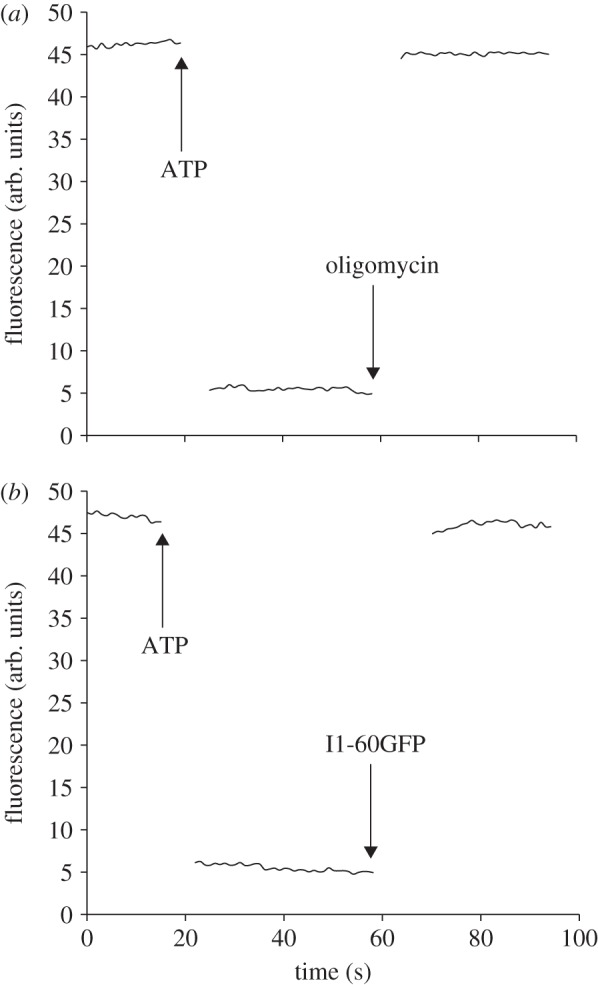
Proton-pumping activity of affinity-purified F_1_F_o_-ATPase reconstituted into liposomes. The purified enzyme had an ATP hydrolase activity of 24.8 µmol min^−1^ mg^−1^, which was 91% sensitive to oligomycin. The formation of a proton-motive force dependent on ATP hydrolysis was monitored from the change of fluorescence of ACMA. The enzyme was inhibited with (*a*) oligomycin and (*b*) the ATPase inhibitor protein, I1–60GFP.

In order to demonstrate that the ATP hydrolase activity of the purified bovine F_1_F_o_-ATPase was coupled to proton pumping, the enzyme purified in the presence of phospholipids (POPC : POPG : POPE, 3 : 1 : 1, by wt) was reconstituted into phospholipid vesicles. The presence of a proton-motive force generated by ATP hydrolysis was demonstrated with the fluorescent probe, ACMA (figure 5). The proton-pumping activity was inhibited by IF_1_ and by oligomycin, inhibitors of the F_1_ and F_o_ domains of the enzyme, respectively.

**Figure 6.**
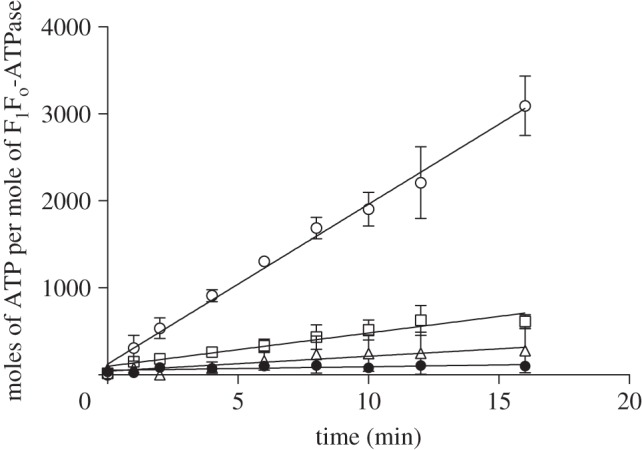
The synthesis of ATP in proteoliposomes containing bovine F_1_F_o_-ATPase and bacteriorhodopsin. The enzyme was purified in the presence of phospholipids (POPC : POPG : POPE, 3 : 1 : 1, by wt) and had a specific activity of 19.3 µmol min^−1^ mg^−1^ that was 92% sensitive to oligomycin. The assay was carried out in the presence of I1–60GFP to inhibit any uncoupled enzyme. Open circles, in the presence of white light: open triangles, in the presence of red light; open squares, in the presence of white light plus FCCP; filled circles, control in the absence of white light and I1–60GFP.

The purified bovine F_1_F_o_-ATPase was also capable of synthesizing ATP. This property was demonstrated with phospholipid vesicles into which the F_1_F_o_-ATPase and bacteriorhodopsin had been co-reconstituted. On illumination with white light, ATP was synthesized in a linear manner, but only in the presence of IF_1_ to prevent ATP hydrolysis by the small amount of uncoupled enzyme that was present in the preparation (figure 6). In the absence of white light, or in the presence of an uncoupler, only traces of ATP were detected. The ATP hydrolysis activity for reconstituted enzyme were 25 and 24.7 µmol min^−1^ mg^−1^ for enzyme purified in the presence and absence of phospholipids, respectively.

## 5. Discussion

The affinity purification of F_1_F_o_-ATPases from mitochondria using the high selectivity of bovine inhibitor protein IF_1_ is a rapid and versatile procedure. As demonstrated here, it provides a ready means of purifying the inhibited and the active enzyme, on a scale compatible with structural analysis, from bovine, ovine, porcine and yeast mitochondria. Given the high conservation of the sequences of IF_1_ in vertebrates, and of the α- and β-subunits with which they interact in the F_1_ domain of the enzyme [40], it is likely that the procedure has a wide applicability for purifying vertebrate F-ATPases. Although the F_1_ sequences are also highly conserved in fungi, the sequences of their IF_1_ proteins are rather less well conserved; yet the bovine IF_1_ provides the means of purifying the yeast F_1_F_o_-ATPase, and the procedure works well with other fungi also (T. J. Charlesworth, I. M. Fearnley, J. V. Bason, M. J. Runswick and J. E. Walker 2013, unpublished data). Thus, the method appears to have general applicability in many multi-cellular and unicellular eukaryotes. A few exceptions have arisen among the invertebrates, but in these cases the use of mutated forms of bovine IF_1_ or the endogenous IF_1_ provides variant avenues that can be explored.

In terms of their chemical purity, as judged by SDS–PAGE analysis and mass spectrometric analysis, the current preparations of the inhibited complex, captured by nickel affinity chromatography using the His-tag at the C-terminus of the I1–60GSTHis, at least equal the purity of earlier preparations purified by a combination of ion exchange and gel filtration chromatography [41]. In both cases, traces of impurities still persist. By contrast, in the most pure active enzyme preparation, where the inhibited complex was captured on a column of immobilized glutathione via the GST domain of the I1–60GSTHis inhibitor, these trace impurities were essentially absent. However, the earlier preparation and the present preparation made in the presence of phospholipids differ most strikingly in their ATP hydrolase activity, and in the coupling of ATP hydrolysis to proton pumping as indicated by the degree of sensitivity of the ATP hydrolase activity to inhibition by oligomycin. The sensitivity of the activity to oligomycin of enzyme that had been affinity-purified in the presence of synthetic phospholipids is comparable with that of the enzyme in vesicles made from the inner membranes of mitochondria (table 1). This same affinity-purified preparation reconstituted into liposomes generated a proton-motive force driven by ATP hydrolysis, and made ATP in the presence of a proton-motive force generated independently by bacteriorhodopsin. This latter experiment could only be made to work by inhibiting the ATP hydrolase activity of the remaining low levels of uncoupled enzyme with the inhibitor protein. The ATP synthesis activity of the reconstituted enzyme (purified in the presence of phospholipids) is 0.36 µmol min^−1^ mg^−1^, approximately 70 times lower than the specific activity of 25 μmol min^−1^ mg^−1^ of ATP hydrolysis by the same reconstituted enzyme. Hence, traces of uncoupled enzyme will mask any synthesis of ATP driven by the proton-motive force. The same demonstration of ATP synthesis by the bovine F_1_F_o_-ATPase co-reconstituted with light-driven bacteriorhodopsin to generate the proton-motive force was influential in establishing Mitchell's chemiosmotic hypothesis [42]. This experiment could only have succeeded with an enzyme preparation that was completely coupled. It should be noted that the enzyme in mitochondrial membranes at the start of the purification has an ATPase activity that is not 100 per cent sensitive to oligomycin (table 1). Therefore, it is possible that a quantity of uncoupled F_1_F_o_-ATPase exists in mitochondrial membranes.

The molecular basis of uncoupling of proton-motive force and hydrolysis of ATP by F_1_F_o_-ATPases has been little discussed and investigated previously. In the 1960s, Kagawa & Racker demonstrated that both the OSCP and F_6_ were required for reconstituting bovine F_1_-ATPase with F_o_ to form a coupled F-ATPase complex [43,44]. In the 1990s, it was realized from other reconstitution experiments [45–47] that the OSCP and F_6_ together with subunits b and d form a separate substructure of the F-ATPase from the central stalk subunits γ, δ and ε. From these observations, the concept emerged of the stator, where the peripheral stalk (subunits b, d, OSCP and F_6_) links the external surface of the F_1_ domain to the a subunit in the F_o_ domain, distinguishing them functionally from the rotor (consisting of the central stalk attached to the c-ring). The limited proteolysis experiments described here add to the molecular understanding of coupling and uncoupling. They illustrate that the structures of parts of the stator domains differ significantly in the uncoupled and coupled F_1_F_o_-ATPase. Uncoupling is accompanied by an increased susceptibility to proteolysis in parts of this structure. Presumably, this effect reflects a change in structure involving the region of contact between the c-ring and subunit a that provides an essential link in the transmembrane proton translocation pathway in the coupled enzyme. It is apparent from current structures of the intact bovine enzyme at 18 Å resolution determined by electron cryomicroscopy [48] that this region of contact is not extensive, but an intact stator is required to maintain the integrity of the transmembrane proton pathway. Increasingly, it is becoming evident that the integrity of this pathway depends upon the presence of bound phospholipids, and especially cardiolipin [10]. Prolonged exposure of the enzyme to detergents, or short-term exposure to relatively high concentrations of many detergents, displaces the bound phospholipids and appears to change the structure of the stator, disrupting the proton pathway and rendering the enzyme uncoupled. In the present rapid-affinity chromatography conducted in the presence of exogenous phospholipids, loss of bound phospholipids is avoided, and the coupling of the enzyme is retained. In future studies, it will be essential to define the bound phospholipids and to understand their roles in maintaining the coupled interface.

## 6. Acknowledgements

J.E.W. designed the research; M.J.R., J.V.B., M.G.M., G.C.R. and I.M.F. carried out the experiments, and together with J.E.W. analysed the data. J.E.W. wrote the paper.

## Supplementary Material

Supplementary data for: The affinity purification and characterization of ATP synthase complexes from mitochondria
