# Supplementary material for: The affinity purification and characterization of ATP synthase complexes from mitochondria
Source: Open Biol. 2013 Feb;3(2):120160. doi: 10.1098/rsob.120160 (PMC3603449; doi:10.1098/rsob.120160)
Supplement: Supplementary data for: The affinity purification and characterization of ATP synthase complexes from mitochondria [file rsob120160-s1.docx]

**Supplementary data for:**

# The affinity purification and characterization of ATP synthase complexes from mitochondria

Michael J. RUNSWICK, John V. BASON, Martin G. MONTGOMERY, Graham C. ROBINSON, Ian M. FEARNLEY and John E. WALKER^1^

The Medical Research Council Mitochondrial Biology Unit, Hills Road, Cambridge CB2 0XY, United Kingdom

^1^To whom correspondence should be sent. Tel: 0044-1223252701; FAX: 0044-1223252705; E-mail: [walker@mrc-mbu.cam.ac.uk](mailto:walker@mrc-mbu.cam.ac.uk).

**Table S1 Masses of mature subunits of bovine, ovine, porcine and yeast F_1_F_o_-ATPases**

| Subunit^*^ |  | Bovine | | |  | Ovine^†‡^ | | |  | Porcine^‡^ | | |  | Yeast | | |
| --- | --- | --- | --- | --- | --- | --- | --- | --- | --- | --- | --- | --- | --- | --- | --- | --- |
|  |  | Calculated | Observed | SD |  | Calculated | Observed | SD |  | Calculated | Observed | SD |  | Calculated | Observed | SD |
| α |  | 55,246.5 | 55,239.9 | 9.1 |  | 55,247.5 | 55,238.3 | 3.6 |  | 55,231.5 | 55,224.2 | 3.6 |  | 54,944.7 | 54,950.3 | 4.9 |
| β |  | 51,705.2 | 51,713.2 | 2.6 |  | 51,503.0 | 51,508.2 | 1.9 |  | 51,563.1 | 51,565.8 | 3.5 |  | 51,126.4 | 51,130.1 | 2.9 |
| γ |  | 30,140.7 | 30,144.6 | 1.3 |  | n.a. | 30,142.8 | 1.6 |  | 30076.7 | 30,078.9 | 1.7 |  | 30,616.2 | 30,618.7 | 1.9 |
| b |  | 24,668.8 | 24,671.0 | 0.7 |  | 24,619.70 | 24,621.7 | 1.1 |  | 24,473.6 | 24,476.0 | 2.0 |  | 23,034.4 | 23,035.3 | 1.3 |
| OSCP |  | 20,929.8 | 20,932.4 | 0.8 |  | 21,013.9 | 21,016.6 | 0.7 |  | 20,888.7 | 20,924.5 | 2.0 |  | 20,871.2 | 20,872.5 | 1.3 |
| d |  | 18,603.3 | 18,605.6 | 0.7 |  | 18,631.4 | 18,632.8 | 5.7 |  | 18,426.3 | 18,427.8 | 0.9 |  | 19,720.5 | 19,721.8 | 0.8 |
| a |  | 24,816.0 | 24,819.0 | 1.3 |  | 24,826.1 | 24,842.2 | 1.3 |  | 25,067.4 | 25,069.5 | 1.5 |  | 27,939.7 | 27,941.8 | 0.8 |
| δ |  | 15,064.9 | 15,067.4 | 1.2 |  | 15,061.0 | 15,062.0 | 1.3 |  | 15,007.8 | 15,008.7 | 0.3 |  | 14,553.5 | 14,554.4 | 0.4 |
| e |  | 8,189.5 | 8,190.4 | 0.7 |  | n.a. | 8,205.6 | 1.5 |  | 8,100.3 | 8,100.9 | 1.6 |  | 10,786.4 | n.d.^§^ |  |
| f |  | 10,208.0 | 10,209 | 2.0 |  | 10,208.0 | 10,208.6 | 1.2 |  | 10,222.0 | 10,221.6 | 0.4 |  | 10,565.2 | 10,565.7 | 0.8 |
| F_6_/h |  | 8,958.1 | 8,958.9 | 0.2 |  | n.a. | 8,990.2 | 1.0 |  | 8,930.0 | 8,930.7 | 0.6 |  | 10,407.4 | 10,407.8 | 0.8 |
| g |  | 11,328.3 | 11,327.2 | 1.6 |  | 11,327.3 | 11,328.2 | 1.8 |  | 11,242.2 | 11,242.9 | 0.9 |  | 12,921.1 | n.d.§ |  |
| A6L |  | 7,964.6 | 7,964.9 | 0.2 |  | 7,938.5 | 7,938.2 | 0.5 |  | 7,947.5 | 7,947.9 | 19.2 |  | 5,850.3 | 5,850.2 | 0.2 |
| ε |  | 5,651.7 | 5,652.5 | 0.6 |  | 5,637.6 | 5,656.0 | 2.4 |  | 5,618.5 | 5,617.6 | 1.3 |  | 6,611.4 | 6,612.1 | 1.9 |
| c |  | 7,650.1 | 7,650.4 | 0.2 |  | 7,650.1 | 7,650.2 | 0.1 |  | 7650.1 | 7,650.19 | 0.2 |  | 7,787.5 | 7,787.7 | 0.1 |

^*^The subunits contain the following stable protein modifications: N-formylation of subunits A6L/aap1, yeast c subunit and mammalian a subunits; N-acetylation of all d and g subunits, mammalian f subunits, and yeast e-subunit, and and N-ε-trimethylation of lysine-43 in mammalian c subunits. ^†^The sequences of the ovine γ, F_6_ and e subunits are not available (n.a.); ^‡^there are unexplained differences between observed and calculated masses for ovine a and ε subunits, and for porcine OSCP; ^§^ n.d., not determined.

**Tables S2-1 to S2-4. Supporting information for the identification of mature subunits of mitochondrial ATP synthase complexes.**

The data were obtained by mass spectrometric analysis of tryptic digests, by peptide mass fingerprinting (PMF) and tandem mass analyses. In almost all cases, proteins were identified with sufficient confidence (p <0.05) by peptide mass data alone, and the tandem mass analysis of peptides provide confirmation. The 95% confidence threshold (p = 0.05) for PMF data corresponds to a score of 76. The ions score is the MASCOT score derived from database comparisons of fragment ions from MS/MS analysis of individual peptide ions. Propionamide arises from reaction of cysteine residues with acrylamide.

**Table S2-1 *S. cerevisiae* ATP synthase**

|  | Peptide Mass Fingerprinting | | | Tandem MS | | | | |
| --- | --- | --- | --- | --- | --- | --- | --- | --- |
| Protein | Peptide  matches | Sequence  Coverage^1^ | Mascot  Score /76 | Obs. mass (MH^+^) | Calc. mass (MH^+^) | Residues^1^ | Sequence | Ions  score |
| α | 20 | 44% | 225 | 1325.7469 | 1325.6808 | 520-530 | SNHNELLTEIR | 81/48 |
|  |  |  |  | 1519.8187 | 1519.7678 | 462-474 | IGEFESSFLSYLK | 88/48 |
|  |  |  |  | 1553.8096 | 1553.7383 | 294-306 | EAYPGDVFYLHSR | 67/48 |
|  |  |  |  | 2073.1680 | 2073.0797 | 63-82 | GMALNLEPGQVGIVLFGSDR | 79/48 |
| β | 16 | 50% | 225 | 1116.6434 | 1116.6411 | 140-149 | VVDLLAPYAR | 47/48 |
|  |  |  |  | 1420.7080 | 1420.6967 | 177-190 | AHGGFSVFTGVGER | 81/48 |
|  |  |  |  | 1677.9355 | 1677.9282 | 43-57 | LVLEVAQHLGENTVR | 82/48 |
|  |  |  |  | 1922.9579 | 1922.9494 | 242-258 | DEEGQDVLLFIDNIFR | 163/48 |
|  |  |  |  | 2260.1846 | 2260.1642 | 273-293 | IPSAVGYQPTLATDMGLLQER | 85/48 |
| γ | 9 | 50% | 380 | 2351.2798 | 2351.1513 | 187-207 | TIEQSPSFGKFEIDTDANVPR | 137/46 |
|  |  |  |  | 2829.6152 | 2829.4345 | 162-186 | ISIFYNDPVSSLSFEPSEKPIFNAK | 107/46 |
| b | 10 | 46% | 505 | 1523.7627 | 1523.7476 | 129-141 | ETVELESEAFELK | 70/48 |
|  |  |  |  | 1632.8562 | 1632.8439 | 105-120 | IDSVSQLQNVAETTK | 61/48 |
|  |  |  |  | 1652.9392 | 1652.9330 | 15-30 | ANSIINAIPGNNILTK | 42/48 |
|  |  |  |  | 1714.9705 | 1714.9585 | 193-207 | VLQQSISEIEQLLSK | 95/48 |
|  |  |  |  | 1903.9983 | 1903.9719 | 105-121 | DRIDSVSQLQNVAETTK | 69/48 |
| OSCP | 8 | 48% | 528 | 1247.6859 | 1247.6994 | 80-90 | NLDGYVVNLLK | 43/46 |
|  |  |  |  | 1325.7025 | 1325.7059 | 68-79 | NSVIDAIVETHK | 61/46 |
|  |  |  |  | 1388.8572 | 1388.8624 | 53-65 | LGHLLLNPALSLK | 95/46 |
|  |  |  |  | 1783.9305 | 1783.9337 | 104-120 | IASDFGVLNDAHNGLLK | 95/46 |
|  |  |  |  | 1873.9613 | 1873.9694 | 12-29 | LFGVEGTYATALYQAAAK | 147/46 |
| d | 9 | 62% | 429 | 1220.6060 | 1220.6521 | 88-97 | QLQVIESFEK | 58/47 |
|  |  |  |  | 1495.7382 | 1495.8002 | 64-76 | NTSVIDKIESYVK | 49/47 |
|  |  |  |  | 2190.0974 | 2190.0826 | 42-59 | QLLELQSQPTEVDFSHYR | 126/47 |
|  |  |  |  | 2834.5547 | 2834.4054 | 116-140 | DLQSTLDNIQSARPFDELTVDDLTK | 67/47 |
| δ | 3 | 34% | 85 | 2154.1580 | 2154.1652 | 119-138 | EAAEAAIQVEVLENLQSVLK | 32/47 |
| ε | 4 | 65% | 68 | 1231.6648 | 1231.6641 | 27-37 | TELQTASVLNR | 91/47 |
|  |  |  |  | 1351.6017 | 1351.6165 | 38-48 | SQTDAFYTQYK | 57/47 |
|  |  |  |  | 1751.9460 | 1751.9438 | 6-22 | AGISYAAYLNVAAQAIR | 140/47 |
|  |  |  |  | 1880.0391 | 1880.0388 | 5-22 | KAGISYAAYLNVAAQAIR | 66/47 |

**Table S2-2 Bovine ATP synthase**

|  | Peptide Mass Fingerprinting | | | Tandem MS | | | | |
| --- | --- | --- | --- | --- | --- | --- | --- | --- |
| Protein | Peptide matches | Sequence coverage^1^ | Mascot Score /76 | Obs. mass (MH^+^) | Calc. mass (MH^+^) | Residues^1^ | Sequence | Ions score |
| α | 16 | 43% | 574 | 1287.6661 | 1287.6943 | 263-273 | HALIIYDDLSK | 85/47 |
|  |  |  |  | 1553.7322 | 1553.7383 | 292-304 | EAYPGDVFYLHSR | 76/47 |
|  |  |  |  | 1624.8848 | 1624.8904 | 91-106 | TGAIVDVPVGEELLGR | 85/47 |
|  |  |  |  | 1667.7690 | 1667.7945 | 46-60 | NVQAEEMVEFSSGLK | 66/47/ |
|  |  |  |  | 2309.1772 | 2309.1594 | 430-460 | QGQYSPMAIEEQVAVIYAGVR | 67/47 |
| β | 19 | 61% | 1010 | 1038.5935 | 1038.5942 | 88-97 | IPVGPETLGR | 61/47 |
|  |  |  |  | 1385.7058 | 1385.7093 | 98-109 | IMNVIGEPIDER | 69/47 |
|  |  |  |  | 1406.6776 | 1406.6811 | 180-193 | AHGGYSVFAGVGER | 90/47 |
|  |  |  |  | 1601.8004 | 1601.8104 | 219-233 | VALVYGQMNEPPGAR | 92/47 |
|  |  |  |  | 1650.8986 | 1650.9173 | 49-63 | LVLEVAQHLGESTVR | 62/47 |
|  |  |  |  | 1921.9540 | 1921.9654 | 249-264 | DQEGQDVLLFIDNIFR | 150/47 |
|  |  |  |  | 1988.0117 | 1988.0335 | 342-360 | R.AIAELGIYPAVDPLDSTSR | 110/47 |
|  |  |  |  | 2266.0745 | 2266.0842 | 279-299 | IPSAVGYQPTLATDMGTMQER | 86/47 |
| γ | 6 | 27% | 360 | 1096.7001 | 1096.6109 | 66-75 | HLIIGVSSDR | 57/48 |
|  |  |  |  | 1213.7198 | 1213.6357 | 76-87 | GLCGAIHSSVAK + propionamide | 49/48 |
|  |  |  |  | 1298.7928 | 1298.6999 | 43-54 | VYGVGSLALYEK | 52/48 |
|  |  |  |  | 1753.0310 | 1752.8949 | 238-252 | NASEMIDKLTLTFNR | 57/48 |
| b | 15 | 73% | 518 | 1077.5519 | 1077.5476 | 122-129 | HYLFDVQR | 57/48 |
|  |  |  |  | 1127.5934 | 1127.6216 | 1-11 | PVPPLPEHGGK | 37/48 |
|  |  |  |  | 1394.7124 | 1394.7096 | 130-141 | NNIAMALEVTYR | 100/48 |
|  |  |  |  | 1506.7249 | 1506.7192 | 155-166 | LDYHISVQNMMR | 68/48 |
|  |  |  |  | 1874.9575 | 1874.9727 | 14-28 | FGLIPEEFFQFLYPK | 42/48 |
| OSCP | 5 | 26% | 244 | 1858.9084 | 1859.0021 | 78-94 | FSPLTSNLINLLAENGR | 84/48 |
|  |  |  |  | 2062.9343 | 2062.9343 | 95-113 | LTNTPAVISAFSTMMSVHR | 61/48 |
| d | 7 | 51% | 85 | 1093.5289 | 1093.5273 | 32-40 | SWNETLTSR | 66/48 |
|  |  |  |  | 1516.8230 | 1516.8158 | 9-21 | TIDWVAFGEIIPR | 92/48 |
|  |  |  |  | 1976.0447 | 1976.0528 | 41-57 | LATLPEKPPAIDWAYYK | 83/48 |
| δ | 1 | 9% | 136 | 1431.7073 | 1431.7074 | 115-128 | AQSELLGAADEATR | 128/45 |
| e | 4 | 46% | 191 | 1176.7163 | 1176.7351 | 1-11 | VPPVQVSPLIK | 73/47 |
|  |  |  |  | 1391.6855 | 1391.7027 | 15-27 | YSALFLGMAYGAK | 30/47 |
| f | 2 | 27% | 98/76 | 1314.7227 | 1314.7238 | 16-26 | LGELPSWILMR | 37/46 |
|  |  |  |  | 1366.6663 | 1366.6750 | 27-39 | DFTPSGIAGAFQR | 43/46 |
| F_6_ | 2 | 44% | 222 | 1773.7778 | 1773.7788 | 53-67 | ADMNTFPNFTFEDPK | 49/47 |
|  |  |  |  | 2032.9063 | 2032.9206 | 23-41 | QTSGGPVDAGPEYQQDLDR | 146/47 |
| g | 4 | 42% | 240 | 1162.5784 | 1162.5931 | 26-34 | LATFWYYAK | 46/47 |
|  |  |  |  | 1557.8776 | 1557.8747 | 11-25 | APALVNAAVTYSKPR | 97/47 |
|  |  |  |  | 2003.1238 | 2003.1423 | 35-53 | VELVPPTPAEIPTAIQSLK | 50/47 |
| A6L | 2 | 31% | 50 | 1597.7604 | 1597.7758 | 31-43 | HNFYHNPELTPTK | 28/47 |

**Table S2-3. Ovine ATP synthase**

Except for subunits A6L, a and c, the sequences of ovine subunits are not in searchable sequence databases. Therefore, the proteins were identified by comparison to the sequences of bovine proteins.

|  | Peptide Mass Fingerprinting | | | Tandem MS | | | | |
| --- | --- | --- | --- | --- | --- | --- | --- | --- |
| Protein ^2^ | Peptide matches | Sequence  coverage^1^ | Mascot  Score /76 | Obs. mass (MH^+^) | Calc. mass (MH^+^) | Residues^1^ | Sequence | Ions score |
| α | 14 | 41% | 177 | 1026.5536 | 1026.5942 | 152-161 | AVDSLVPIGR | 69/47 |
|  |  |  |  | 1553.6776 | 1553.7383 | 292-304 | EAYPGDVFYLHSR | 69/47 |
|  |  |  |  | 1624.8221 | 1624.8904 | 91-106 | TGAIVDVPVGEELLGR | 65/47 |
|  |  |  |  | 2169.0483 | 2169.1451 | 464-482 | FENAFLSHVISQHQALLSK | 70/47 |
|  |  |  |  | 2309.0757 | 2309.1594 | 430 450 | QGQYSPMAIEEQVAVIYAGVR | 74/47 |
| β | 17 | 52% | 254 | 1038.5336 | 1038.5942 | 88-97 | IPVGPETLGR | 77/48 |
|  |  |  |  | 1385.6396 | 1385.7093 | 98-109 | IMNVIGEPIDER | 83/48 |
|  |  |  |  | 1406.6112 | 1406.6811 | 180-193 | AHGGYSVFAGVGER | 51/48 |
|  |  |  |  | 1601.7332 | 1601.8104 | 219-233 | VALVYGQMNEPPGAR | 49/48 |
|  |  |  |  | 1921.8790 | 1921.9654 | 249-264 | DQEGQDVLLFIDNIFR | 148/48 |
|  |  |  |  | 1987.9387 | 1988.0335 | 342-360 | AIAELGIYPAVDPLDSTSR | 69/48 |
|  |  |  |  | 2265.9924 | 2266.0842 | 279-299 | IPSAVGYQPTLATDMGTMQER | 76/48 |
| γ | 7 | 31% | 75 | 1096.6183 | 1096.6109 | 66-75 | HLIIGVSSDR | 50/48 |
|  |  |  |  | 1213.6263 | 1213.6357 | 76-87 | GLCGAIHSSVAK + propionamide | 49/48 |
|  |  |  |  | 1752.9042 | 1752.8949 | 238-252 | NASEMIDKLTLTFNR | 85/48 |
| b | 5 | 26% | 162 | 1077.5239 | 1077.5476 | 122-129 | HYLFDVQR | 50/49 |
|  |  |  |  | 1394.6769 | 1394.7096 | 130-141 | NNIAMALEVTYR | 49/49 |
| OSCP | 6 | 35% | 260 | 1288.6786 | 1288.6752 | 154-165 | IDPSIMGGMIVR | 53/47 |
|  |  |  |  | 1859.0125 | 1859.0021 | 78-94 | FSPLTSNLINLLAENGR | 119/47 |
| d | 7 | 50% | 361 | 1093.5273 | 1093.5273 | 32-40 | SWNETLTSR | 44/47 |
|  |  |  |  | 1516.8204 | 1516.8158 | 9-21 | TIDWVAFGEIIPR | 106/47 |
|  |  |  |  | 1976.0389 | 1976.0528 | 41-57 | LATLPEKPPAIDWAYYK | 88/47 |
|  |  |  |  | 2493.2217 | 2493.2217 | 123-143 | NIIPFDQMTIEDLNEVFPETK | 123/47 |
| δ | 1 | 9% | 122 | 1431.7372 | 1431.7074 | 115-128 | AQSELLGAADEATR | 115/47 |
| e | 5 | 63% | 222 | 1176.7219 | 1176.7351 | 1-11 | VPPVQVSPLIK | 60/47 |
| f | 2 | 27% | 78 | 1366.6812 | 1366.6750 | 27-39 | DFTPSGIAGAFQR | 40/47 |
| F_6_ | 2 | 44% | 120 | 2032.9264 | 2032.9206 | 23-41 | QTSGGPVDAGPEYQQDLDR | 108/48 |
| A6L (ovine) | 3 | 31% | 84 | 1631.7557 | 1631.7635 | 31-41 | HNFYHNPELMTTK | 50/48 |
| g | 2 | 41% | 150 | 1234.7020 | 1234.6790 | 26-34 | LATFWYYAK | 35/46 |
|  |  |  |  | 2367.1433 | 2367.2626 | 11-25 | APALVNAAVTYSKPR | 85/46 |
| ε | 3 | 46% | 171 | 907.5081 | 907.4996 | 6-13 | QAGLSYIR | 54/48 |
|  |  |  |  | 951.5201 | 951.5145 | 24-31 | DALKTEFK | 58/48 |
| c (ovine) | 1 | 41% | 30 | 2810.5227 | 2810.5198 | 8-38 | FIGAGAATVGVAGSGAGIGTVFGSLIIGYAR | 25/45 |

**Table S2-4. Porcine ATP synthase**

|  | Peptide Mass Fingerprinting | | | Tandem MS | | | | |
| --- | --- | --- | --- | --- | --- | --- | --- | --- |
| Protein | Peptide matches | Sequence  coverage^1^ | Mascot  Score /76 | Obs. mass (MH^+^) | Calc. mass (MH^+^) | Residues^1^ | Sequence | Ions score |
| α | 15 | 43% | 577 | 1553.7354 | 1553.7383 | 292-304 | EAYPGDVFYLHSR | 88/46 |
|  |  |  |  | 1624.8818 | 1624.8904 | 91-106 | TGAIVDVPVGEELLGR | 87/46 |
|  |  |  |  | 1667.7672 | 1667.7945 | 46-60 | NVQAEEMVEFSSGLK | 68/46 |
|  |  |  |  | 2139.1243 | 2139.1345 | 464-482 | FENAFLSHVISQHQALLGK | 108/46 |
|  |  |  |  | 2338.1594 | 2338.1673 | 399-420 | EVAAFAQFGSDLDAATQQLLSR | 41/46 |
| β | 18 | 56% | 1060 | 1385.7195 | 1385.7093 | 98-109 | IMNVIGEPIDER | 70/48 |
|  |  |  |  | 1406.6904 | 1406.6811 | 180-193 | AHGGYSVFAGVGER | 91/48 |
|  |  |  |  | 1601.8142 | 1601.8104 | 219-233 | VALVYGQMNEPPGAR | 108/48 |
|  |  |  |  | 1650.9108 | 1650.9173 | 49-63 | LVLEVAQHLGESTVR | 68/48 |
|  |  |  |  | 1815.8660 | 1815.8694 | 361-376 | IMDPNIVGSEHYDVAR | 119/48 |
|  |  |  |  | 1921.9696 | 1921.9654 | 249-264 | DQEGQDVLLFIDNIFR | 139/48 |
|  |  |  |  | 1988.0243 | 1988.0335 | 342-360 | AIAELGIYPAVDPLDSTSR | 89/48 |
|  |  |  |  | 2266.0920 | 2266.0842 | 279-299 | IPSAVGYQPTLATDMGTMQER | 93/48 |
| γ | 7 | 29% | 390 | 1096.6116 | 1096.6109 | 66-75 | HLIIGVSSDR | 84/47 |
|  |  |  |  | 1213.6255 | 1213.6357 | 76-87 | GLCGAIHSSVAK + propionamide | 64/47 |
|  |  |  |  | 1312.7500 | 1312.7147 | 43-54 | VYGIGSLALYEK | 84/47 |
|  |  |  |  | 1322.6534 | 1322.6739 | 119-129 | THSDQFLVTFK | 46/47 |
|  |  |  |  | 1752.8984 | 1752.8949 | 238-252 | NASEMIDKLTLTFNR | 92/47 |
|  |  |  |  | 1982.0170 | 1982.0269 | 203-218 | NYKEYSLANIIYYSLK | 91/47 |
| b | 7 | 16% | 427 | 1077.6201 | 1077.5476 | 122-129 | HYLFDVQR | 57/47 |
|  |  |  |  | 1233.7181 | 1233.6487 | 121-129 | RHYLFDVQR | 55/47 |
|  |  |  |  | 1394.7949 | 1394.7096 | 130-141 | NNIAMALEVTYR | 115/47 |
|  |  |  |  | 1522.7881 | 1522.7141 | 155-166 | LDYHISVQNMMR | 76/47 |
| OSCP | 8 | 56% | 565 | 1274.6603 | 1274.6596 | 154-165 | VDPSIMGGMIVR | 63/48 |
|  |  |  |  | 1858.9272 | 1859.0021 | 78-94 | FSPLTSNLINLLAENGR | 112/47 |
|  |  |  |  | 2007.9938 | 2007.9991 | 95-113 | LSSTPGVISAFSTMMSVHR | 88/48 |
|  |  |  |  | 2116.0728 | 2116.1396 | 76-94 | EKFSPLTSNLINLLAENGR | 169/47 |
| d | 11 | 77% | 624/94 | 1093.5321 | 1093.5273 | 32-40 | SWNETLTSR | 51/63 |
|  |  |  |  | 1486.8254 | 1486.8043 | 9-21 | AIDWVAFGEIIPR | 81/63 |
|  |  |  |  | 1765.8756 | 1765.8603 | 85-99 | YTALVDAEEQEDVKR | 139/63 |
|  |  |  |  | 1946.0533 | 1946.0422 | 41-58 | LAALPEKPPAIDWAYYK | 86/63 |
| a | 1 | 6% | 37/77 | 1658.9610 | 1658.9476 | 42-55 | TISIQQWLIQLTSK | 18/46 |
| δ | 2 | 20% | 128 | 1417.7130 | 1417.6917 | 115-128 | AQSELLGAADEASR | 72/59 |
|  |  |  |  | 3341.6919 | 3341.6007 | 1-34 | AEAAAAPASAAGPGQMSFTFASPTQVFFNGANVR | 74/46 |
| e | 4 | 45% | 172 | 953.6058 | 953.5203 | 29-35 | YNYLKPR | 25/47 |
|  |  |  |  | 1176.8191 | 1176.7351 | 1-11 | VPPVQVSPLIK | 58/47 |
|  |  |  |  | 1359.8384 | 1359.7307 | 15-27 | YSALFLGVAYGAK | 22/47 |
| F_6_ | 3 | 57% | 230 | 1773.7617 | 1773.7788 | 53-67 | ADMNTFPNFTFEDPK | 39/47 |
|  |  |  |  | 2032.9277 | 2032.9206 | 23-41 | QTSGGPVDAGPEYQQDLDR | 139/47 |
| f | 2 | 27% | 85 | 1314.7250 | 1314.7238 | 16-26 | LGELPSWILMR | 46/48 |
|  |  |  |  | 1366.6802 | 1366.6750 | 27-39 | DFTPSGIAGAFQR | 34/48 |
| g | 4 | 52% | 311 | 1136.5930 | 1136.5888 | 26 -34 | LATFWHYAK | 55/61 |
|  |  |  |  | 1585.9190 | 1585.9061 | 11-23 | APVLVNAAVTYSKPR | 81/61 |
|  |  |  |  | 2003.1309 | 2003.1423 | 35-53 | VELVPPTPAEIPTAIQSLK | 63/47 |
| A6L | 3 | 50% | 129 | 1015.4568 | 1015.4666 | 47-54 | HSTPWEMK | 46/46 |
|  |  |  |  | 1194.7900 | 1194.7609 | 58-67 | IYLPLLLPPR | 59/45 |
| ε | 1 | 16% | 40/77 | 907.5590 | 907.4996 | 6-13 | QAGLSYIR | 27/42 |
| c | 1 | 40% | 50/77 | 2810.3470 | 2810.5198 | 8-38 | FIGAGAATVGVAGSGAGIGTVFGSLIIGYAR | 31/45 |

**
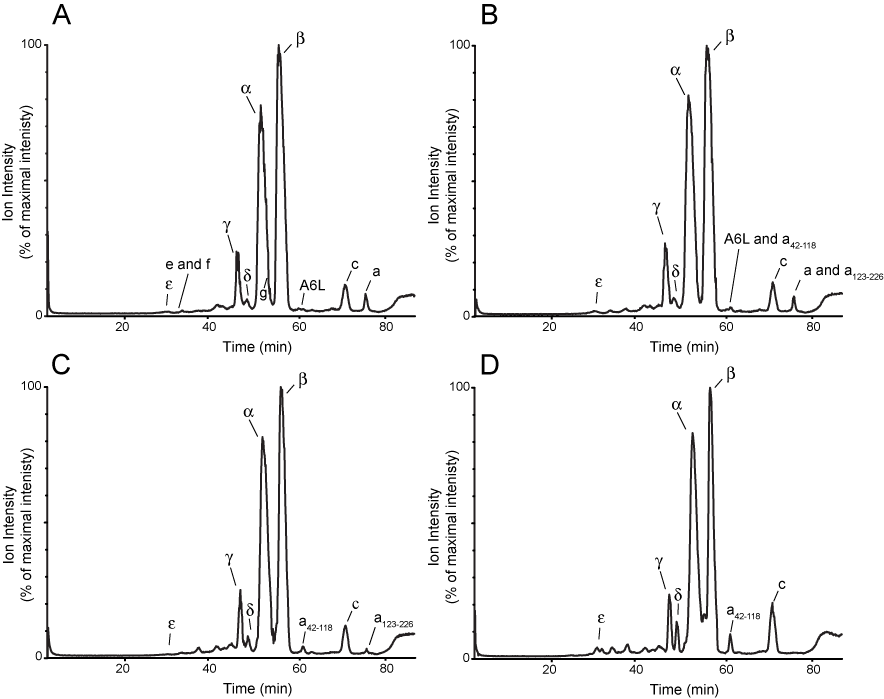
**

**Figure S1 LC-MS analysis of the products of partial trypsinolysis of the hydrophobic and F_1_-subunits subunits of uncoupled bovine F_1_F_o_-ATPase**

This analysis Panels A, B, C and D represent the analysis of samples taken after 0, 20, 40 and 120 min of trypsinolysis, respectively. The samples were precipitated with ethanol. The precipitate was solubilised in a mixture of formic acid:trifluoroethanol:hexafluoro-isopropanol:water (60.0:15.0:1.0:24.0, by vol.) and analysed by LC-MS. The traces show the total ion current of the column eluent measured in an electrospray triple quadrupole mass spectrometer. The elution positions of hydrophobic and F_1_-subunits are indicated.


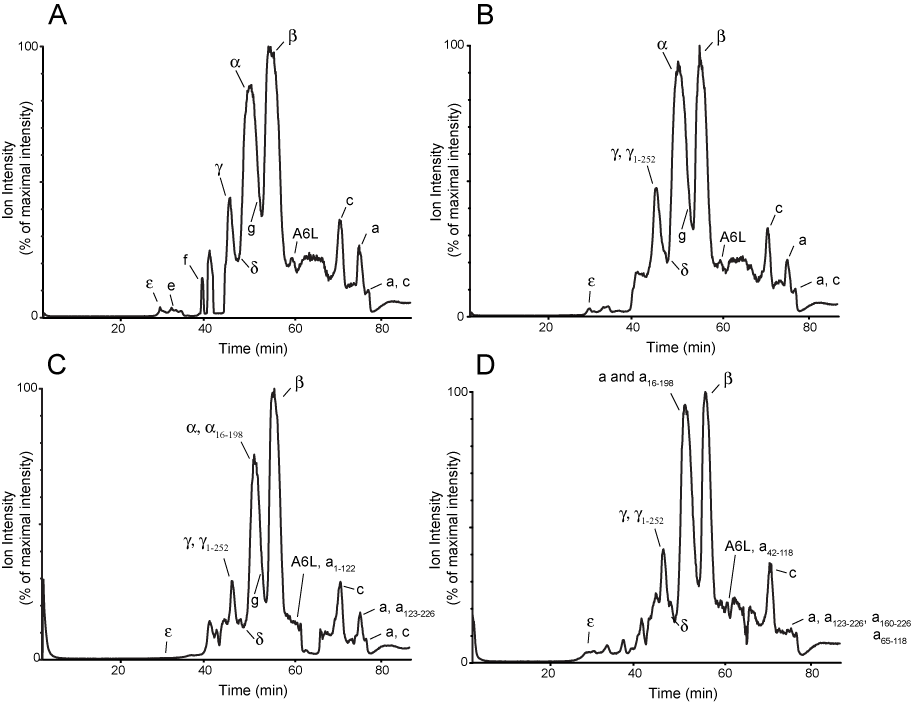


**Figure S2 LC-MS analysis of the products of partial trypsinolysis of the hydrophobic and F_1_-subunits of coupled bovine F_1_F_o_-ATPase**

Panels A, B, C and D represent the analysis of samples taken after 0, 20, 40 and 120 min of trypsinolysis, respectively. For details see the legend to Fig S1.


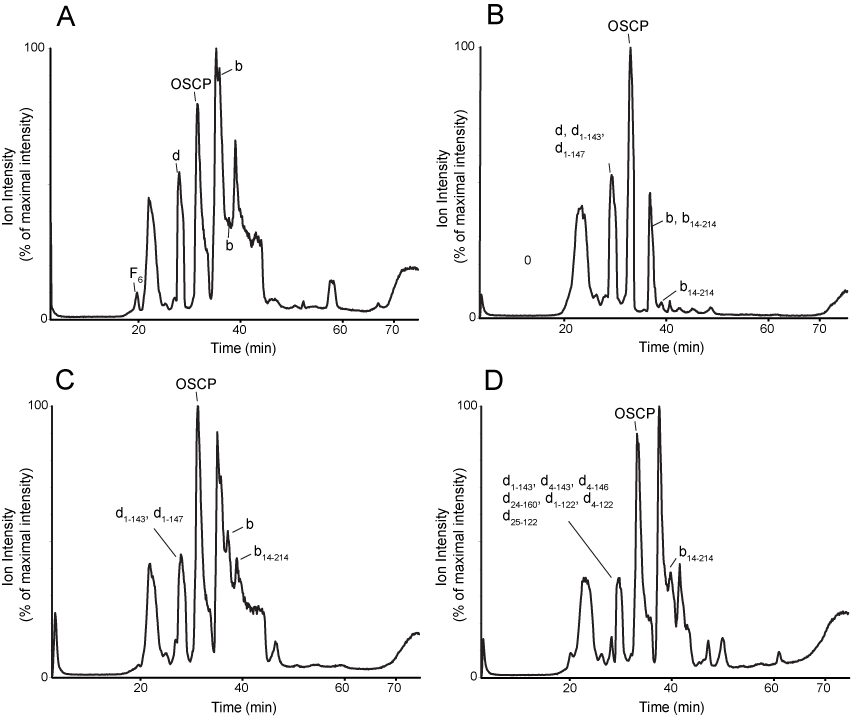


**Figure S3 LC-MS analysis of the products of partial trypsinolysis of the peripheral stalk subunits of uncoupled bovine F_1_F_o_-ATPase**

Panels A, B, C and D represent samples taken after 0, 20, 40 and 120 min of trypsinolysis, respectively. Samples were acidified with trifluoroacetic acid, and hexafluoro-isopropanol and trifluoroethanol were added to final concentrations of 1% and 15%, respectively. The traces show the total ion current of the column eluent measured in an electrospray triple quadrupole mass spectrometer. The elution positions of the peripheral stalk subunits of F_1_F_o_-ATPase and their proteolytic products are shown.

**
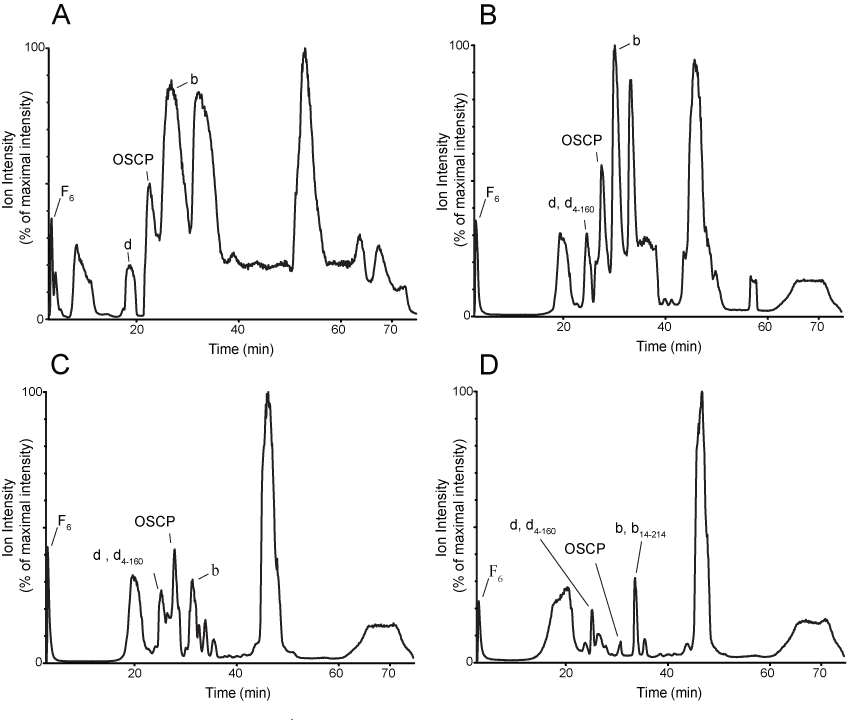
**

**Figure S4 LC-MS analysis of the products of partial trypsinolysis of the peripheral stalk subunits of coupled bovine F_1_F_o_-ATPase**

Panels A, B, C and D represent samples taken after 0, 20, 40 and 120 min of trypsinolysis, respectively. For further details see the legend to Fig. S3. The elution positions of the peripheral stalk subunits of F_1_F_o_-ATPase and their proteolytic products are shown.

**Table S3** **Analysis by LC-MS of subunits and fragments of these subunits from bovine F_1_F_o_-ATPase produced by mild trypsinolysis of the uncoupled enzyme**

|  | Mass (Da) | | Time (min) | |  |
| --- | --- | --- | --- | --- | --- |
| Protein | Observed | Calculated | First observed | Last observed | Explanation |
| a^1^ | 24, 818.1 | 24, 816.0 | 0 | 20 | Intact subunit |
| a^3^ | 11, 170.2 | 11, 169.6 | 10 | 60 | Residues 123-226 |
| a^4^ | 8, 595.2 | 8, 594.4 | 20 | 120 | Residues 42-118 |
| A6L^1^ | 7, 964.5 | 7, 964.6 | 0 | 20 | Intact subunit |
| b^1^ | 24, 669.2 | 24, 668.8 | 0 | 60 | Intact subunit |
| b^2^ | 23, 304.1 | 23, 303.9 | 10 | 120 | Residues 14-214 |
| c^1^ | 7, 650.2 | 7, 650.1 | 0 | 120 | Intact subunit |
| d^1^ | 18, 603.6 | 18, 603.3 | 0 | 40 | Intact subunit |
| d^2^ | 16, 437.0 | 16, 437.4 | 10 | 120 | Residues 1-143 |
| d^3^ | 16, 921.1 | 16, 921.7 | 10 | 60 | Residues 1-147 |
| d^4^ | 16, 110.9 | 16, 110.4 | 60 | 120 | Residues 4-143 |
| d^5^ | 16, 466.8 | 16, 466.8 | 120 | 120 | Residues 4-146 |
| d^6^ | 15, 982.4 | 15, 982.3 | 120 | 120 | Residues 24-160 |
| d^7^ | 13, 960.3 | 13, 961.0 | 120 | 120 | Residues 1-122 |
| d^8^ | 13, 635.4 | 13634.6 | 120 | 120 | Residues 4-122 |
| d^9^ | 11, 209.8 | 11, 211.7 | 120 | 120 | Residues 25-122 |
| e^1^ | 8, 190.0 | 8, 189.5 | 0 | 0 | Intact subunit |
| f^1^ | 10, 208.6 | 10, 208.1 | 0 | 0 | Intact subunit |
| F_6_^1^ | 8, 958.6 | 8, 958.1 | 0 | 10 | Intact subunit |
| g^1^ | 11, 328.0 | 11, 328.3 | 0 | 10 | Intact subunit |
| OSCP^1^ | 20, 929.3 | 20, 929.8 | 0 | 120 | Intact subunit |
| OSCP^2^ | 14, 565.9 | 14, 561.3 | 10 | 120 | Residues 51-184 |
| OSCP^3^ | 6, 618.1 | 6, 616.8 | 120 | 120 | Residues 7-65 |

**Table S3 cont**.

|  | Mass (Da) | | Time (min) | |  |
| --- | --- | --- | --- | --- | --- |
| Protein | Observed | Calculated | First observed | Last observed | Explanation |
| α^1^ | 55, 254.9 | 55, 246.5 | 0 | 120 | Intact subunit |
| α^2^ | 19, 472.8 | 19, 470.4 | 10 | 120 | Residues 16-198 |
| β^1^ | 51, 711.7 | 51, 705.2 | 0 | 120 | Intact subunit |
| γ^1^ | 30, 144.0 | 30, 140.7 | 0 | 120 | Intact subunit |
| δ^1^ | 15, 065.7 | 15, 064.9 | 0 | 120 | Intact subunit |
| ε^1^ | 5, 651.7 | 5, 651.7 | 0 | 120 | Intact subunit |

**Table S4** **Analysis by LC-MS of subunits and fragments of these subunits from bovine F_1_F_o_-ATPase produced by mild trypsinolysis of the coupled enzyme**

|  | Mass (Da) | | Time (min) | |  |
| --- | --- | --- | --- | --- | --- |
| Protein | Observed | Calculated | First observed | Last observed | Explanation |
| a^1^ | 24, 818.1 | 24, 816.0 | 0 | 120 | Intact subunit |
| a^2^ | 13, 664.6 | 13, 664.4 | 40 | 120 | Residues 1-122 |
| a^3^ | 11, 169.3 | 11, 169.6 | 40 | 120 | Residues 123-226 |
| a^4^ | 8, 594.6 | 8, 594.4 | 40 | 120 | Residues 42-118 |
| a^5^ | 7187.0 | 7, 186.7 | 120 | 120 | Residues 160-226 |
| a^6^ | 5, 834.0 | 5, 834.0 | 120 | 120 | Residues 65-118 |
| A6L^1^ | 7, 964.3 | 7, 964.6 | 0 | 120 | Intact subunit |
| c^1^ | 7, 650.2 | 7, 650.1 | 0 | 120 | Intact subunit |
| d^1^ | 18, 603.1 | 18, 603.3 | 0 | 60 | Intact subunit |
| d^2^ | 18, 277.2 | 18, 277.9 | 20 | 120 | Residues 4-160 |
| e^1^ | 8, 190.2 | 8, 1895 | 0 | 0 | Intact subunit |
| f^1^ | 10, 207.9 | 10, 208.1 | 0 | 0 | Intact subunit |
| F_6_^1^ | 8, 958.3 | 8, 958.1 | 0 | 60 | Intact subunit |
| g^1^ | 11, 327.6 | 11, 328.3 | 0 | 40 | Intact subunit |
| OSCP^1^ | 20, 929.3 | 20, 929.8 | 0 | 120 | Intact subunit |
| OSCP^2^ | 14, 564.4 | 14, 561.3 | 10 | 120 | Residues 51-184 |
| OSCP^3^ | 6, 617.6 | 6, 616.8 | 120 | 120 | Residues 7-65 |
| α^1^ | 55, 244.5 | 55, 246.5 | 0 | 120 | Intact subunit |
| α^2^ | 19, 470.8 | 19, 470.4 | 20 | 120 | Residues 16-198 |
| β^1^ | 51, 710.2 | 51, 705.2 | 0 | 120 | Intact subunit |
| γ^1^ | 30, 141.4 | 30, 140.7 | 0 | 120 | Intact subunit |
| γ^2^ | 28, 061.5 | 28, 061.2 | 0 | 120 | Residues 1-252 |
| δ^1^ | 15, 066.1 | 15, 064.9 | 0 | 120 | Intact subunit |
| ε^1^ | 5, 651.4 | 5, 651.7 | 0 | 120 | Intact subunit |
